# Effects of Information Architecture on the Effectiveness and User Experience of Web-Based Patient Education in Middle-Aged and Older Adults: Online Randomized Experiment

**DOI:** 10.2196/15846

**Published:** 2021-03-03

**Authors:** Tessa Dekkers, Marijke Melles, Stephan B W Vehmeijer, Huib de Ridder

**Affiliations:** 1 Faculty of Industrial Design Engineering Delft University of Technology Delft Netherlands; 2 Faculty of Behavioural, Management and Social sciences University of Twente Enschede Netherlands; 3 Department of Orthopaedic Surgery Reinier de Graaf Hospital Delft Netherlands

**Keywords:** user-computer interface, total joint replacement, user-centered design, health education, mobile phone, computer-assisted instruction, patient education as topic, models, theoretical, middle aged, aged, humans, internet

## Abstract

**Background:**

Web-based patient education is increasingly offered to improve patients’ ability to learn, remember, and apply health information. Efficient organization, display, and structural design, that is, information architecture (IA), can support patients’ ability to independently use web-based patient education. However, the role of IA in the context of web-based patient education has not been examined systematically.

**Objective:**

To support intervention designers in making informed choices that enhance patients’ learning, this paper describes a randomized experiment on the effects of IA on the effectiveness, use, and user experience of a patient education website and examines the theoretical mechanisms that explain these effects.

**Methods:**

Middle-aged and older adults with self-reported hip or knee joint complaints were recruited to use and evaluate 1 of 3 patient education websites containing information on total joint replacement surgery. Each website contained the same textual content based on an existing leaflet but differed in the employed IA design (tunnel, hierarchical, or matrix design). Participants rated the websites on satisfaction, engagement, control, relevance, trust, and novelty and completed an objective knowledge test. Analyses of variance and structural equation modeling were used to examine the effects of IA and construct a theoretical model.

**Results:**

We included 215 participants in our analysis. IA did not affect knowledge gain (*P*=.36) or overall satisfaction (*P*=.07) directly. However, tunnel (mean 3.22, SD 0.67) and matrix (mean 3.17, SD 0.69) architectures were found to provide more emotional support compared with hierarchical architectures (mean 2.86, SD 0.60; *P*=.002*)*. Furthermore, increased perceptions of personal relevance in the tunnel IA (β=.18) were found to improve satisfaction (β=.17) indirectly. Increased perceptions of active control in the matrix IA (β=.11) also improved satisfaction (β=.27) indirectly. The final model of the IA effects explained 74.3% of the variance in satisfaction and 6.8% of the variance in knowledge and achieved excellent fit (*χ*^2^_17,215_=14.7; *P*=.62; root mean square error of approximation=0.000; 95% CI [0.000-0.053]; comparative fit index=1.00; standardized root mean square residual=0.044).

**Conclusions:**

IA has small but notable effects on users’ experiences with web-based health education interventions. Web-based patient education designers can employ tunnel IA designs to guide users through sequentially ordered content or matrix IA to offer users more control over navigation. Both improve user satisfaction by increasing user perceptions of relevance (tunnel) and active control (matrix). Although additional research is needed, hierarchical IA designs are currently not recommended, as hierarchical content is perceived as less supportive, engaging, and relevant, which may diminish the use and, in turn, the effect of the educational intervention.

## Introduction

### Background

Verbal and written patient education methods are often supplemented with web-based education to improve patients’ ability to learn, remember, and apply health information. Such improvements are needed because patients’ recall of traditional education is generally poor [1-3], which negatively affects their satisfaction with care, ability to self-manage, and emotional well-being [4,5].

There are many options to engage patients with web-based education, ranging from animations and interactive exercises to tailored health advice [6]. However, for education to be the most effective, patients must be able to use such functions independently. An efficient information architecture (IA) supports independent use [7,8], yet few studies have systematically examined IA in the context of web-based health education. To support intervention designers in making informed choices that enhance patients’ learning, this paper describes a randomized experiment concerning the effect of IA on the effectiveness, use, and user experience of a patient education website and the theoretical mechanisms that explain these effects. In addition, the study explores the benefit of tailoring IA to specific user profiles.

### IA

IA concerns “the structural design of a shared information environment” [9]. It describes “the way in which digital content is organized and displayed, which strongly impacts users’ ability to find and use content” [10]. IA has a pervasive role in website design because it affects the user’s ability to find information with no or very limited training and helps save long-term costs. Web-based environments with effective IAs are typically more scalable, easier to maintain and update, and require fewer redesigns [9]. Yet, despite the importance of IA, there is a lack of primary research that examines IA specifically in the context of web-based health education. A recent review on this subject revealed that to date, only 1 study has empirically manipulated IA in isolation from other design features [10]. This study, conducted in 2012 by Crutzen et al [11] to examine web-based hepatitis information, investigated whether providing users with the opportunity to skip pages (or not) affected website use and user perceptions of efficiency, effectiveness, and enjoyment. It was found that an architecture that provided users with less control over navigation increased both website use and knowledge gain [11]. Although this study demonstrated that IA influences web-based learning experiences, it examined only one particular IA design (the tunnel). Therefore, we argue that a more comprehensive examination of IA is required. For this purpose, we used the taxonomy of 4 archetypes of IA by Danaher et al [12,13]: tunnel, hierarchical, matrix, and hybrid architectures. Hybrid architectures mix design elements of tunnel, hierarchical, and matrix architectures. Each hybrid mix may thereby present unique advantages and disadvantages that cannot be readily understood before experimentation with the nonhybrid IA designs. Therefore, this study focuses on the three nonhybrid IA designs (ie, tunnel, hierarchical, and matrix) only. The features, advantages, and disadvantages of each design are outlined below, and additional examples of each IA design are presented in the *Methods* section of this paper.

The tunnel IA design is the most common IA in health interventions: 90%-100% of interventions for chronic illness or mental health support include some form of tunneling [14]. In a typical tunnel, IA users follow a step-by-step approach to access content in a predefined, sequential order. For example, a website that only allows access to new material once users have completed previous lessons can be considered to have a tunneled design. A possible advantage of this IA is that it reduces the complexity of information. However, it also reduces the perceived control of users, which may decrease engagement and lead to nonadherence and attrition [15]. The second IA archetype is the hierarchical design. Hierarchical designs organize content hierarchically, differentiating between major and minor content. Typically, users are first provided with a general overview of the major content present on the website. For example, the official United States government website on health organizes content by major topics such as “Health Insurance,” “Medications,” and “Vaccines and Immunizations.” After selecting the appropriate topic, users can explore nested, minor content to review in detail. Assumed advantages of this IA include increased control over content selection, familiarity, and simplicity. However, usability may be limited when users are unable to locate deeply nested content. The third IA concerns the matrix design. This IA design presents all available content on 1 home page or dashboard, thereby removing any differentiation between major and minor content or predefined sequential paths included in the hierarchical and tunnel designs, respectively. This allows users to freely navigate content in their preferred order and duration. Travel agency websites that display all available travel options first and then allow users to sort on date, price, or location are examples of matrix designs. The matrix IA design is considered engaging yet disorienting and is particularly appropriate for highly educated and experienced users looking for enrichment [15,16].

### What Explains the Effects of IA?

**Figure 1:**
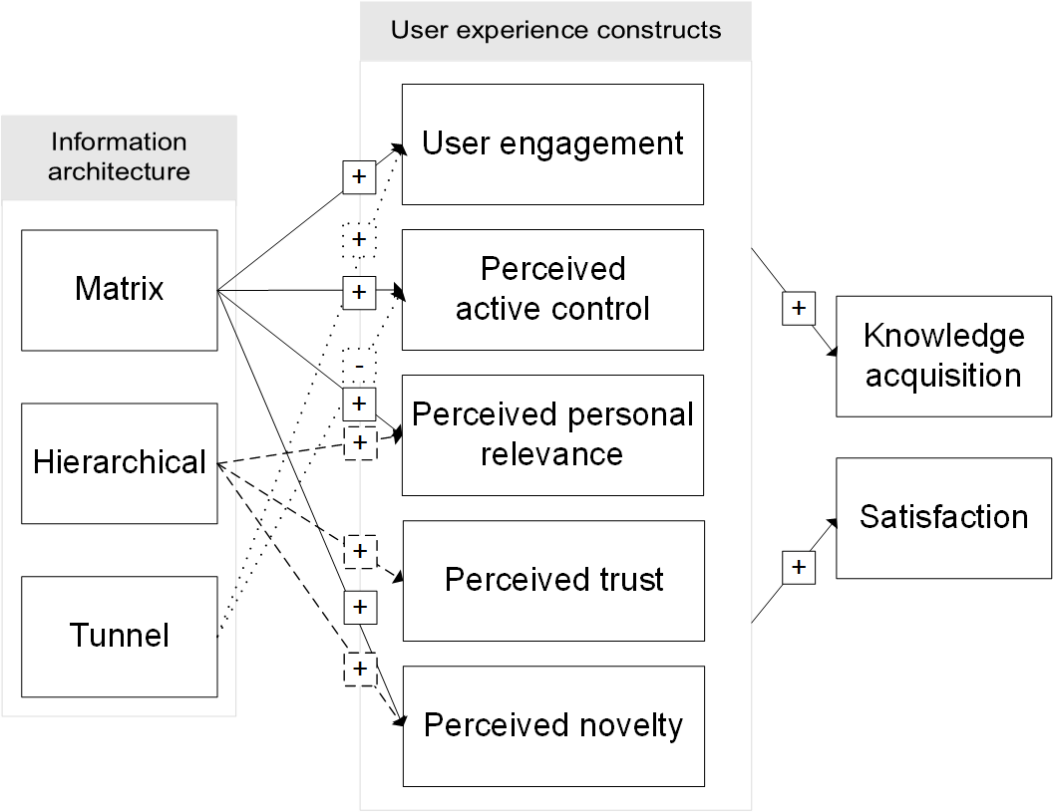
Conceptual model of information architecture (IA). Solid arrows represent expected effects related to matrix IA design, dashed arrows represent expected effects related to hierarchical IA design, and dotted arrows represent expected effects related to tunnel IA design.

Many scholars have condemned the *black box* approach to eHealth, which offers little understanding of the underlying mechanisms through which web-based interventions (and the tools, techniques, and strategies embedded in them) exert their effects [13,14,17]. IA design has the same issue. Although there are several assumed benefits (eg, increased usability and increased user control) of each IA design, as outlined above, there is no overarching conceptual model of IA effects. This makes it difficult to determine how IA affects the user experience of a health education website. Therefore, we examine the following 5 aspects of the user experience: user engagement, user perceptions of control, personal relevance, trustworthiness, and novelty, which may be influenced by IA design in depth. These are depicted in the conceptual model (Figure 1). We do not hold specific expectations regarding the main effects of IA design but rather expect that each IA design may elicit a different user experience in comparison with the other IA designs, as detailed below.

#### User Engagement

First, we hypothesize that IA design affects user engagement. User engagement is defined as “a quality of user experience characterized by the depth of an actor’s investment when interacting with a digital system” [18,19]. It is often conceptualized as a multidimensional construct composed of cognitive, affective, and behavioral components [20], which means that engagement can both refer to a subjective experience of flow and immersion as well as the actual act of using an intervention [15]. Several recent reviews suggest that user engagement is pivotal for creating an effective and enjoyable web-based experience [15,21].

Our expectations regarding IA design as a determinant of engagement are twofold. First, tunnel IA designs (in comparison with hierarchical and matrix IA) are thought to increase behavioral engagement because the sequential, predefined setup allows researchers to persuasively guide users through the web-based process, resulting in extended use [11,14]. In a study of a web-based smoking cessation intervention, users who viewed content in a set order accessed content more often and for longer [22]. This indicates that tunnel IA design should result in higher levels of behavioral user engagement. In contrast, a more flexible matrix IA design may increase the subjective experience of engagement by providing the user more control over the interaction, as outlined below.

#### Perceived Active Control

As stated earlier, tunnel IA designs have been found to decrease user perceptions of control [11]. User control is a “user’s ability to voluntarily participate in and instrumentally influence a communication” [23,24]. As matrix IA designs allow users to both influence the selection of content and the order in which content is consumed, this design is expected to increase perceptions of user control. Active user control is a dimension of interactivity [23,24], and interactive interventions, in turn, are associated with a more engaging experience [6,25]. Possibly, this is because users who are able to influence an intervention instrumentally consider this to be an enjoyable experience or become more emotionally invested in the intervention. It is important to note here that *perceived* interactivity and control appear to be more important than actual website interactivity [26,27]. Together, this indicates that matrix IA designs may also improve (cognitive or affective components of) engagement through increased user perceptions of control.

#### Perceived Personal Relevance

Perceived personal relevance refers to the extent to which people feel that information is relevant to themselves and their situation [28-30]. People are more motivated to process personally relevant content, leading to deeper processing and greater susceptibility to any persuasive attempts the content makes [28,31,32]. Perceptions of relevance have also been linked to educational enjoyment [33]. We expect that perceived personal relevance may increase knowledge acquisition through the same motivational pathway. Hierarchical and matrix IA designs are the only designs that allow users to select content. We expect that users, to some extent, select content based on what they consider most personally relevant. Therefore, we hypothesize that hierarchical and matrix IA designs (in contrast to tunnel IA design) increase the perceived personal relevance of the health information presented and that this leads to both greater knowledge acquisition and greater satisfaction.

#### Perceived Trust

Perceived trust is a belief that influences whether a patient is willing to engage with health education [34]. Trust in health information is influenced by source, message, channel, and recipient [35,36] as well as structural website features [37]. A previous study on the credibility of health websites showed that the presence of a navigation menu (as is included in most hierarchical IA designs) increases perceived website credibility, as it reinforces the notion that the website is produced by a professional organization [37]. This type of heuristic evaluation of information credibility can lead to a better experience on the health website [38]. Therefore, we hypothesize that hierarchical IA design positively influences participants’ trust in the health information presented and, in turn, the knowledge and satisfaction derived from the education.

#### Perceived Novelty

Finally, we considered perceived novelty as a potential explanatory variable. As the tunnel IA design is the norm in health interventions, other IA designs may offer more novel ways to access health information. Novelty in the context of interfaces can “act as a curiosity generating mechanism that arouses the imaginations of users and captures their interest in a site” [39]. Users pay greater attention and effort to novel media [40], subsequently leading to a greater uptake of information. Novelty has also been related to enjoyable experiences of flow and engagement [18,38]. Therefore, we expect that the less common IA designs (hierarchical and matrix) will increase user perceptions of novelty and that increased novelty will improve both user satisfaction and knowledge acquisition through increased attention to the content.

### Does One IA Design Fit All?

A final consideration in examining the effects of IA is the role of individual preferences and capabilities. Many recommendations regarding IA design take user characteristics into account. For example, Lynch and Horton [16] describe matrix IA designs (which they refer to as *webs*) as more suitable for highly educated users with a high level of prior knowledge about the content. It has also been suggested that perceived control over website navigation may be more important to some users than to others [11]. However, the influence of individual differences on the effectiveness and experience of different IA designs has not been empirically tested.

This study used a previously defined set of user profiles of patients [41] who had undergone total joint replacement (TJR) surgery to explore the potential benefit of tailored IA design (Table 1). Each profile represents 1 of 3 ways through which communicative preferences and capabilities may manifest in patients. So-called *managing* patients prefer open, participative communication, particularly regarding personal circumstances, and have high capabilities and self-efficacy for understanding and applying health information. In comparison, *optimistic* patients have similar capabilities but find patient-provider communication of lesser importance and only have a slight preference for an open communicative style. Finally, *modest* patients value both open information and emotional support but have limited self-efficacy and skills in health communication. With these profiles and the recommendations for each IA design in mind, we hypothesize that users with higher preferences for open communication (ie, managing patients) will prefer IA designs that offer more control (ie, matrix), optimistic patients will not prefer any IA design in particular, and modest patients will prefer more supportive IA designs that guide them through the educational content step by step (ie, tunnel).

**Table 1:** Description of communicative preferences and capabilities of three total joint replacement patient profiles^a^.

Managing profile	Optimistic profile	Modest profile
High preference for open communication	Moderate preference for open communication	Moderate preference for open communication
High preference for emotionally supportive communication	Low preference for emotionally supportive communication	Moderate preference for emotionally supportive communication
High critical communication capabilities	Moderate critical communication capabilities	Low critical communication skills
High personal communication capabilities	Moderate personal communication capabilities	Low personal communication skills
High self-efficacy for health information	High self-efficacy for health information	Low self-efficacy for health information

^a^Patient profiles are based on Groeneveld et al [41].

### Study Objectives

The aims of this study are threefold: (1) to test the effects of IA in the context of a TJR surgery patient education website on knowledge acquisition and satisfaction with web-based education; (2) to test possible working mechanisms of IAs, including user engagement, perceived user control, perceived personal relevance, perceived trust, and perceived novelty; and (3) to explore the potential of tailored IAs.

## Methods

### Design

In July 2018, we conducted a between-subjects experiment comparing the knowledge and satisfaction gained from a patient education website with three different IA designs. Ethics approval for this study was obtained from the Human Research Ethics Committee Delft University of Technology. Participants provided written consent and signed a data processing agreement formulated in concordance with the General Data Protection Regulation.

### Participants and Procedure

Participants were recruited using a Dutch web-based consumer research service (respondenten.nl B.V.). Middle-aged to older adults (40-80 years) with self-reported chronic hip or knee joint complaints (including arthrosis, wear and tear, chronic inflammation, birth deficits, or unknown causes) were eligible for participation. To detect a small-to-medium effect (*f*^2^=0.15-0.25) on satisfaction and knowledge using an α of .05 and a power of 0.80, a sample size between 159 and 432 participants was needed [42,43]. We aimed to recruit at least 100 participants per condition for a total sample of 300 participants. In total, we were able to enroll 235 participants, of which the data of 215 participants were included in the analysis (see the *Results* section). Participants received monetary reimbursement (15 euro [US $18.2]) for their participation.

The complete experiment was conducted on the web via survey software (Qualtrics). Each eligible participant was provided a hyperlink to the survey. After providing consent, participants filled out questionnaires regarding their communication preferences and skills, health, anxiety, and coping behavior, which were used to determine the patient profile [41]. Participants also stated the extent to which they already felt knowledgeable about TJR surgery (part A). In part B, participants were randomly assigned to 1 of 3 experimental conditions using Qualtrics’ built-in randomizer. The allocation sequence and assignments were concealed from all participants, the researchers, and the consultant hired for participant recruitment until all data were collected. Participants were initially asked to focus on either the website’s design or its content. After reviewing the website’s design, participants reported satisfaction and user perceptions. They were then asked to view the website for a second time while focusing on content. Then, they completed a knowledge test designed for the purpose of this study. The order of focus (design vs content) was counter-balanced. Finally, participants shared their sociodemographic information and received a code for reimbursement (part C). Eligible participants who had not started or completed the survey after 3 weeks were reminded via email once.

### Materials

#### Design Process

The three websites were designed between March and June 2018 by a design agency (Panton B.V.) specializing in the design of products, services, and processes for health care under the supervision of the first author. The lead designer provided literature on IA [12] and was given access to patient profile role descriptions and anonymized data about patients’ communication preferences and capabilities collected in an earlier study (T Dekkers, PhD, unpublished data, February 2017). In June, prototypes of the websites were pilot tested. To discuss progress and ensure accuracy and quality of health information shared on the patient education websites, the design team met with the first author 10 times throughout the design process. At 2 points in the design process (after first conceptualization and after the pilot tests), the design team also met with the full research team, including an orthopedic surgeon.

#### Pilot Usability Study

Prototypes of the three websites were pilot tested with 7 patients (age range 46-77 years) scheduled for TJR surgery and 7 informal caregivers (age range 42-76 years) in June 2018. The pilot test focused specifically on usability of the websites rather than effectiveness in terms of knowledge acquisition. Interested patients present at the clinic for scheduled group-based patient education were shown the prototypes after they provided written consent. They first freely explored the websites while mentioning aloud any (positive or negative) aspects that stood out. Then, they were asked to find information about the first checkup after surgery. This assignment was used to identify usability issues and software bugs [44]. Finally, patients were asked to report engagement using the User Engagement Scale-Short Form (UES-SF, see *Measurements* section). Throughout the pilot test, the cursor of the participants was tracked using screen capture software (CamStudio Recorder v2.7, Rendersoft Development). Screen captures were used both to identify unclear navigational cues and to get an initial impression of whether the users navigated through the IAs as intended (eg, whether patients explored more pages in the matrix design, made use of the table of contents in the hierarchical design, and moved step by step using the next and prior buttons in the tunnel design). The input of patients and caregivers was shared with the lead designer and implemented in the following iteration of the design. This led to significant improvements in usability, including less scrollable text, more prominently displayed contact information, vivid color accents, and larger buttons.

#### Websites

All websites contained the same textual content based on an existing patient education leaflet titled *Instructions after an outpatient Total Hip Prosthesis (THP; Instructies na een Totale Heup Prothese [THP] in dagbehandeling)* used by the local hospital (Reinier de Graaf Gasthuis, the Netherlands). The leaflet addressed practical concerns before and after outpatient THP surgery, including preparation for surgery, pain, medication, and physiotherapy. All graphic design elements (including photos, fonts, and color) were equivalent across websites.

**Figure 2:**
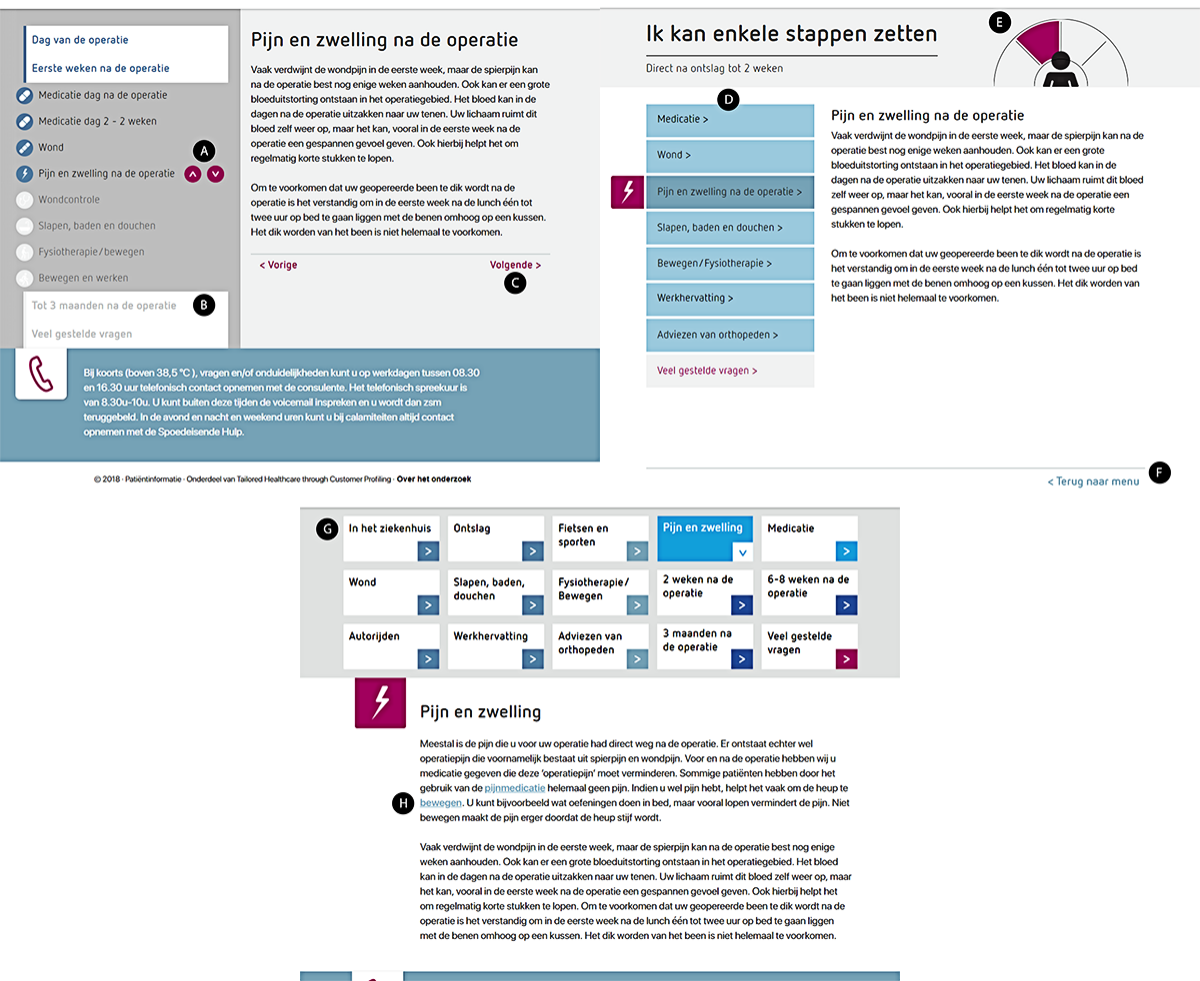
Annotated screenshots of tunnel, hierarchical, and matrix information architecture (IA) design of a Dutch patient education website to prepare patients for total joint replacement surgery. Tunnel IA: (A) next/previous buttons, (B) grayed-out text (not yet accessible), and (C) next/previous buttons. Hierarchical IA: (D) table of contents, (E) major grouping by recovery phase, and (F) return to main menu. Matrix IA: (G) topic matrix and (H) hyperlink. All screenshots depict the same content about pain and swelling (pijn en zwelling).

The tunnel IA website design had a chronological sequential ordering of topics presented as a timeline, starting with *the day of the operation* and ending with the *3-month follow-up* and *frequently asked questions*. Navigation was limited to *next* and *previous* buttons placed below the text and in the timeline. Topics that were not yet accessible to the user were grayed out (Figure 2). The hierarchical IA website design presented participants with a choice menu in which they selected the phase of their *patient journey* (eg, in the hospital and able to walk a few steps). After selecting an option, users were presented with topics grouped in a table-of-content menu. Participants could further investigate their chosen topic using the menu and could return to the home page using the buttons or navigation path (ie, *bread crumb trail*). The matrix IA website design showed all topics in tiles on the home page and provided no suggested reading order. By clicking on the topic tiles or hyperlinks in the body of text, participants could switch between topics. Offline copies of the experimental websites are available on request by contacting the first author.

### Measurements

The primary outcomes of interest are knowledge acquisition and website satisfaction. Satisfaction with web-based education captures both the attitude of patients toward website functioning (eg, satisfaction with comprehensibility and with emotional support derived from the website) as well as their affective attitude (eg, satisfaction with website attractiveness) [45,46]. The secondary outcomes used to test the conceptual model include user perceptions of engagement, control, personal relevance, trust, and novelty. We also measured use by capturing the total time spent on the website in minutes. Finally, we collected short qualitative feedback forms on the perceived advantages and disadvantages of the website.

#### Knowledge Acquisition and Satisfaction With Website

A total of 5 multiple-choice (MC) questions and 3 open questions about (self-)care after TJR surgery were used to assess knowledge acquisition. The questions were based on the information provided on the websites and included, for example: *after the surgery, it is important to strengthen the muscles surrounding the hip joint. Which ways to do so are recommended by orthopedic surgeons?* Each question included the following answer options: *not been discussed*, *discussed, but I cannot remember the details*, a correct answer, and an incorrect answer (distractor) [47]. For each correct MC answer, participants scored 1 point, and for each open question, an answer sheet was developed that assigned points from 0 (incorrect), 1 (partly correct), to 2 (fully correct). All points were summed and converted to reflect the percentage of correct answers (0%-100% correct).

Satisfaction with patient education was measured using the Website Satisfaction Scale [45,46] comprising three subscales: satisfaction with the (1) attractiveness, (2) comprehensibility of the information, and (3) emotional support received from the website. All items consisted of statements to which participants’ agreement was measured on a 5-point Likert scale (1=*totally disagree* and 5=*totally agree*). Statements included *the website looks nice*, *the website is understandable*, and *the website give ease of mind*. Both the overall index score of satisfaction and the separate subscales achieved excellent reliability (α=.82-.98).

#### User Perceptions of Engagement, Active Control, Personal Relevance, Trust, and Novelty

We included 5 constructs to explore the theoretical mechanisms through which (tailored) IAs may influence knowledge acquisition and satisfaction. The first is user engagement, as measured through the UES-SF [19]. We obtained permission to translate this validated questionnaire to Dutch according to the guidelines for cross-cultural adaptation of self-reported instruments [48,49] (personal communication by HL O’Brien, May 18, 2018). The instrument contains 12 questions, which form 1 index score (α=.88), and 4 subscales: focused attention (*I was absorbed in this experience*, α=.75), aesthetic appeal (*the website was attractive*, α=.87), reward (*using the website was worthwhile*, α=.71), and perceived usability (*I felt frustrated while using the website*, α=.79; Multimedia Appendix 1 [18,19,50]). The other user perceptions of interest included perceived active control (*during the website visit, I could freely decide what I wanted to see*, 4 items, α=.96) [27], personal relevance (*the website was relevant to my situation*, 2 items, α=.83) [51], trust (*the website is sincere and honest*, 3 items, α=.97) [34], and novelty (*the website incited my curiosity*, 3 items, α=.90) [50]. All questions were answered on a 5-point Likert scale (1=*strongly disagree* and 5=*strongly agree*).

### Statistical Methods

We conducted chi-square (χ^2^) and analyses of variance (ANOVA) tests to check whether background characteristics were evenly distributed over experimental conditions. To test the main effect of IA, 2 ANOVA tests were conducted with satisfaction and knowledge gain as dependent variables. Follow-up pairwise *t* tests were performed to explore differences between the IA designs, and these were all corrected using the Bonferroni correction. Finally, ANOVA tests were performed with the secondary outcomes (user perceptions) as dependent variables, and the concept of tailored IAs was explored in a two-way ANOVA with condition and profile as the independent variables.

To construct a conceptual model of how IA influences satisfaction and knowledge acquisition, we used structural equation modeling. User perceptions of engagement, personal relevance, active control, trust, and novelty (hereafter, mediating variables) were regressed on IA. Satisfaction and knowledge were regressed on IA and the mediating variables. To improve the parsimony and fit of the model, we removed nonsignificant paths. As our hypotheses suggest that IA design may influence perceived control and subsequently user engagement, and ultimately satisfaction and knowledge, we also constructed a separate serial mediation model for this hypothesis specifically. Model chi-square (χ^2^), comparative fit index (CFI), standardized root mean square residual (SRMR), and root mean square error of approximation (RMSEA) were used to determine model fit. A model was considered to have a good fit when *χ*^2^ divided by degrees of freedom ≤3 with *P*<.05, CFI≥0.95, SRMR≤0.09, and RMSEA≤0.07 [52,53]. All analyses were conducted using R version 3.5.1 [54] with α=.05.

## Results

### Participant Characteristics

**Figure 3:**
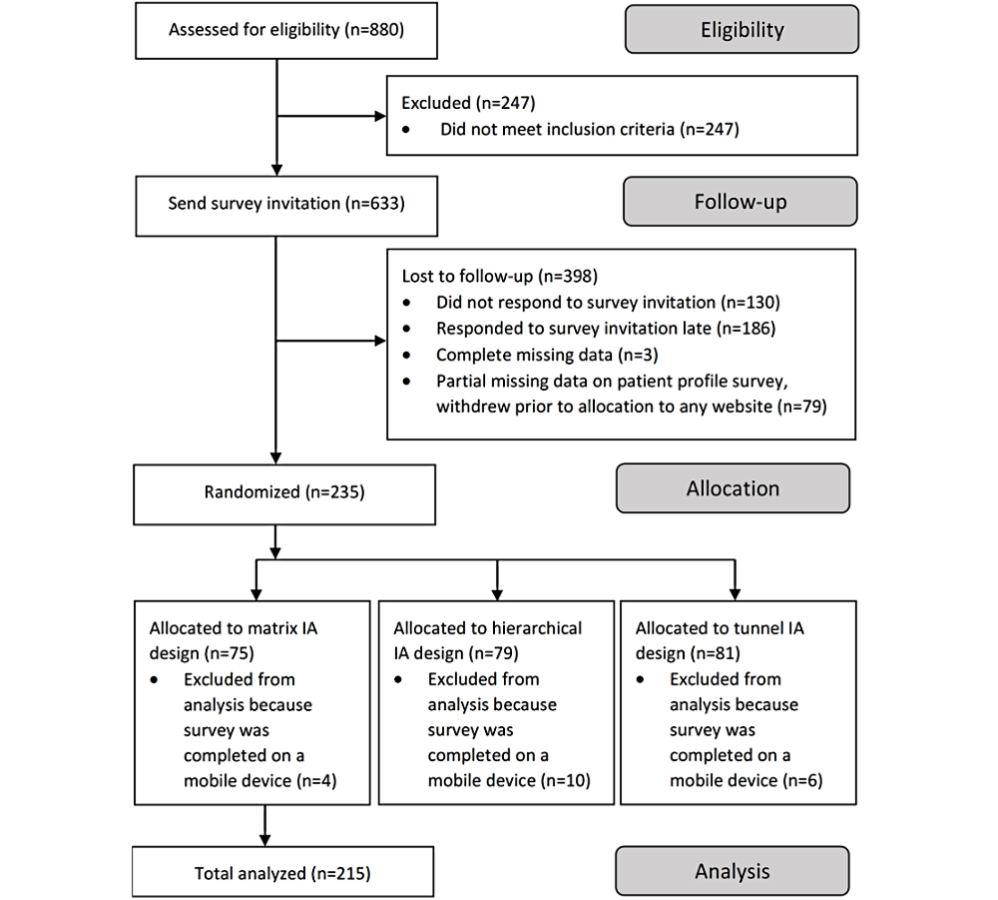
Participant recruitment and follow-up diagram. IA: information architecture.

We enrolled 235 participants, of which, 215 participants were included in the analysis (Figure 3). A total of 20 participants completed the survey on a mobile device, despite instructions to view the survey and the websites on a laptop or personal computer. As the layout and, thus, the information architecture of the websites may appear distorted on mobile devices, these participants were excluded from analysis. There were no significant differences between the excluded participants compared with the included participants with respect to background characteristics, except for device use (*P*<.001). Excluded participants used the personal computer less (47% vs 9% nonuse) and tablet devices more (89% vs 41% use). No significant associations were found between background characteristics and experimental conditions, indicating that participants were evenly distributed over all three conditions. All participant characteristics are reported in Table 2. On average, participants were 57 years old (SD 7.7), female (155/215, 72.1%), attained lower secondary education (95/215, 44.2%), and were employed or self-employed (118/215, 54.9%). They used the internet daily (mean 3.2 hours, SD 2.1) mainly on personal computers or laptops (91%) and mobile phones (82%). Participants rated their overall health significantly lower (69 out of 100) than the Dutch average of 81.5 for people aged 50-59 years [55,56] and experienced considerable movement-evoked joint pain (mean 4.9, SD 2.3).

**Table 2:** Participant characteristics (N=215).

Variable		Value
Age (years), mean (SD)^a^		57.18 (7.70)
**Sex, n (%)**		
	Female	155 (72.1)
	Male	60 (27.9)
**Education, n (%)**		
	Primary education	3 (1.4)
	Lower secondary education	95 (44.2)
	Higher secondary education	36 (16.7)
	Tertiary education	81 (37.7)
**Occupation, n (%)**		
	Employed	83 (38.6)
	Self-employed	35 (16.3)
	Retired	37 (17.2)
	Beneficiary	29 (13.5)
	Other or none	31 (14.4)
**Relationship status, n (%)**		
	Married or long-term relationship	132 (61.4)
	Divorced	41 (19.1)
	Never married	35 (16.3)
	Widowed	5 (2.3)
	Other	2 (0.9)
**Social support^b^, n (%)**		
	Partner	124 (57.7)
	Friend	75 (34.9)
	Child	52 (24.2)
	Neighbor	36 (16.7)
	Family member	34 (15.8)
	Colleague	7 (3.3)
	Group (church or sports)	4 (1.9)
	Other	2 (0.9)
	No support	25 (11.6)
Internet use in hours per day, mean (SD)^c^		3.17 (2.14)
**Device use^b^, n (%)^a^**		
	Personal computer or laptop	194 (90.7)
	Phone	175 (81.8)
	Tablet	88 (41.1)
Self-reported previous knowledge of hip replacement surgery, mean (SD)^d^		1.85 (0.92)
**Patient profile, n (%)**		
	Optimistic	90 (41.9)
	Modest	72 (33.5)
	Managing	53 (24.7)

^a^Data were missing for 1 participant.

^b^Participants could select multiple answers.

^c^Data were missing for 10 participants.

^d^Data were missing for 2 participants.

### Effects of IA on Knowledge Acquisition and Satisfaction

**Figure 4:**
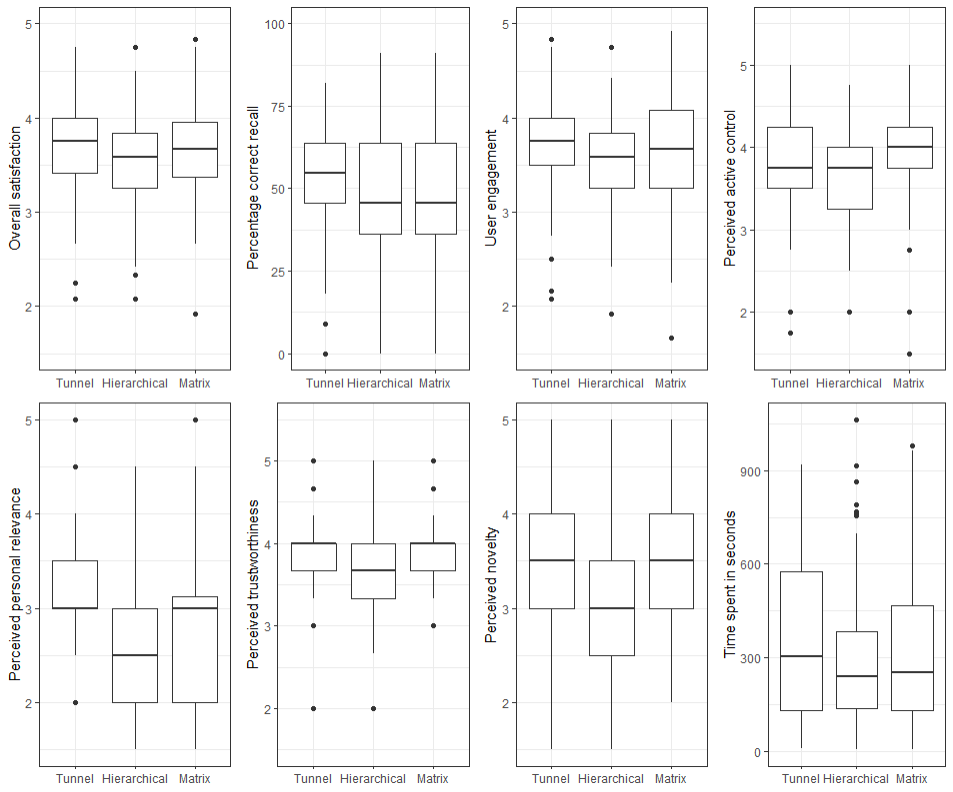
Main effects of information architecture.

All three websites received predominantly positive feedback via the open qualitative feedback forms; participants appreciated that they were *clear and organized*. Multimedia Appendix 2 summarizes the qualitative feedback on advantages and disadvantages for each IA. Table 3 and Figure 4 report the overall effects of IA. IA did not directly affect knowledge acquisition (*F*_2,212_=1.023; *P*=.36; η_p_^2^=0.010) or overall satisfaction (*F*_2,212_=2.702; *P*=.07; η^2^=0.025). IA did have a significant effect on satisfaction with emotional support (*F*_2,212_=6.376; *P*=.002; η^2^=0.057). Post hoc analyses indicated that participants were significantly less satisfied with the hierarchical IA design (mean 2.86, SD 0.60) compared with the matrix (mean 3.17, SD 0.69) and tunnel (mean 3.22, SD 0.67) architectures. The hierarchical design was perceived as the least favorable in general: users devoted less focused attention (mean difference to tunnel −0.31; *P*=.03), saw the design as less novel (mean difference to tunnel −0.33; *P*=.02 and mean difference to matrix −0.36; *P*=.01) and less personally relevant (mean difference to tunnel −0.44; *P*=.006), and found that it provided the least active control (mean difference to matrix −0.32; *P*=.02).

**Table 3:** Knowledge acquisition, satisfaction, and user perceptions of patient education website by information architecture.

Outcome, mean (SD)		Tunnel IA^a^ (n=75)	Matrix IA (n=71)	Hierarchical IA (n=69)	*P* value	(η^2^)^b^
**Website satisfaction**		3.69 (0.52)	3.65 (0.52)	3.50 (0.48)	.07	N/A^c^
	Attractiveness	3.73 (0.61)	3.68 (0.65)	3.61 (0.61)	.50	N/A
	Comprehension	4.24 (0.56)	4.21 (0.59)	4.17 (0.71)	.79	N/A
	Emotional support	3.22 (0.67)	3.17 (0.69)	2.86 (0.60)	.002^d^	.057
Knowledge acquisition		51.64 (19.55)	48.02 (19.75)	47.3 (19.63)	.36	N/A
**User engagement**		3.71 (0.55)	3.65 (0.55)	3.48 (0.57)	.047^e^	.028
	Focused attention	3.16 (0.75)	3.00 (0.70)	2.85 (0.79)	.04^f^	.030
	Esthetic appeal	3.76 (0.68)	3.75 (0.68)	3.52 (0.76)	.08	N/A
	Reward	3.81 (0.62)	3.78 (0.57)	3.58 (0.68)	.06	N/A
	Perceived usability	4.08 (0.68)	4.05 (0.78)	3.98 (0.78)	.67	N/A
Perceived active control		3.84 (0.67)	3.95 (0.65)	3.63 (0.74)	.02^g^	.035
Perceived personal relevance		3.08 (0.86)	2.73 (0.83)	2.64 (0.86)	.005^h^	.050
Perceived trustworthiness		3.94 (0.56)	3.92 (0.57)	3.78 (0.59)	.21	N/A
Perceived novelty		3.43 (0.75)	3.46 (0.73)	3.10 (0.76)	.007^i^	.046
Time spent in minutes:seconds		5:53 (4:24)	5:18 (4:15)	4:59 (4:09)	.44	N/A

^a^IA: information architecture.

^b^Effect size is only provided for significant differences.

^c^N/A: not applicable.

^d^Hierarchical IA was significantly different from both tunnel IA (*P*=.02) and matrix IA (*P*=.02).

^e^Hierarchical IA was significantly different from tunnel IA (*P*=.05).

^f^Hierarchical IA was significantly different from tunnel IA (*P*=.03).

^g^Hierarchical IA was significantly different from matrix IA (*P*=.02).

^h^Tunnel IA was significantly different from both hierarchical IA (*P*=.006) and matrix IA (*P*=.04).

^i^Hierarchical IA was significantly different from both tunnel IA (*P*=.03) and matrix IA (*P*=.01).

### Model of IA Effects

The ANOVA tests demonstrated that the tunnel and matrix designs performed significantly better than the hierarchical IA design. To explain why tunnel and matrix IAs perform better compared with hierarchical IAs, we selected the hierarchical IA as the reference category in the mediation model.

The first mediation model (Model 1) specified that the effect of IA on knowledge and satisfaction would be mediated by user perceptions of engagement, active control, personal relevance, trust, and novelty. Specification of complete mediation results in a fully saturated regression model with zero degrees of freedom, as the number of observations is equal to the number of parameters [57,58]. Therefore, the first model was interpreted based on the regression paths instead of the fit indices (Table 4). All pathways (of which the exact *P* values are provided in Table 4) with *P*<.10 were considered in a second model (Model 2). For Model 3 and Model 4, we continued eliminating pathways with a more stringent cut-off of *P*<.05.

**Figure 5:**
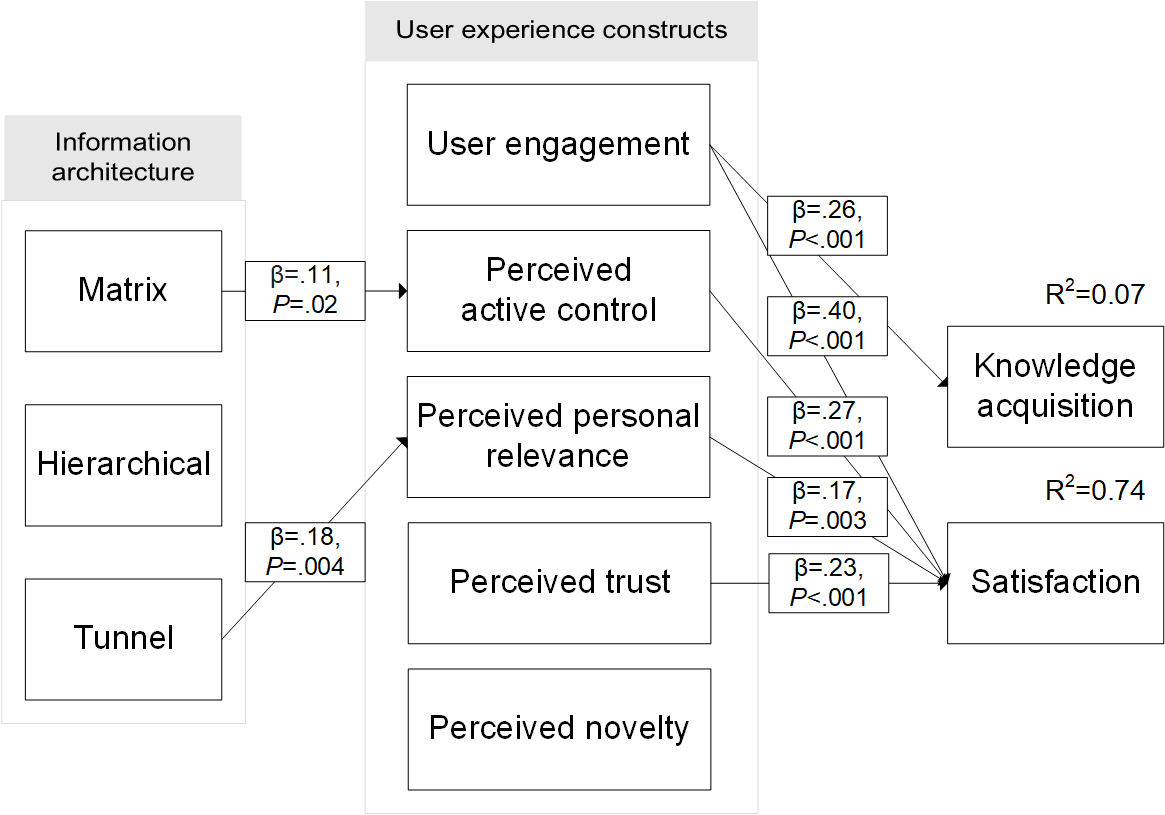
Structural equation model of the effects of information architecture.

Overall, models 2 to 4 all achieved similarly good fit (Table 5). Model 4 (Figure 5) was selected as the final model, as it was the most parsimonious (expressed by highest degrees of freedom [59]). This model explained 74.3% of the variance in satisfaction and 6.8% of the variance in knowledge and achieved excellent fit (*χ*^2^_17,215_=14.7; *P*=.62; RMSEA=0.000; CI 0.000-0.053; CFI=1.00; SRMR=0.044).

**Table 4:** Pathways included in mediation models 1, 2, 3, and 4.

Outcome and predictor or mediator		Path estimate (Model 1)	*P* value (Model 1)	Model 2	Model 3	Model 4
**User engagement**						
	Tunnel IA^a^	0.190	.02	✓^b^	✓	—^c^
	Matrix IA	0.139	.08	✓	—	—
**Perceived active control**						
	Tunnel IA	0.142	.07	✓	—	—
	Matrix IA	0.215	.006	✓	✓	✓
**Perceived personal relevance**						
	Tunnel IA	0.243	.002	✓	✓	✓
	Matrix IA	0.048	.54	—	—	—
**Trust**						
	Tunnel IA	0.133	.09	✓	—	—
	Matrix IA	0.109	.17	—	—	—
**Perceived novelty**						
	Tunnel IA	0.208	.007	✓	—	—
	Matrix IA	0.225	.004	✓	✓	—
**Knowledge**						
	User engagement	0.226	.045	✓	✓	✓
	Perceived active control	0.006	.96	—	—	—
	Perceived personal relevance	0.089	.22	—	—	—
	Trust	−0.007	.93	—	—	—
	Perceived novelty	−0.006	.95	—	—	—
**Satisfaction**						
	User engagement	0.382	<.001	✓	✓	✓
	Perceived active control	0.273	<.001	✓	✓	✓
	Perceived personal relevance	0.169	<.001	✓	✓	✓
	Trust	0.227	<.001	✓	✓	✓
	Perceived novelty	0.026	.60	—	—	—
**Knowledge**						
	Tunnel IA design	0.042	.59	—	—	—
	Matrix IA design	−0.018	.82	—	—	—
**Satisfaction**						
	Tunnel IA design	−0.011	.80	—	—	—
	Matrix IA design	−0.017	.68	—	—	—
**Knowledge**						
	User engagement×matrix IA	0.031	.19	—	—	—
	Perceived novelty×matrix IA	−0.001	.95	—	—	—
	Trust×matrix IA	−0.001	.93	—	—	—
	Perceived personal relevance×matrix IA	0.004	.58	—	—	—
	Perceived active control×matrix IA	0.001	.96	—	—	—
	User engagement×tunnel IA	0.043	.12	—	—	—
	Perceived novelty×tunnel IA	−0.001	.95	—	—	—
	Trust×tunnel IA	−0.001	.93	—	—	—
	Perceived personal relevance×tunnel IA	0.022	.25	—	—	—
	Perceived active control×tunnel IA	0.001	.96	—	—	—
**Satisfaction**						
	User engagement×tunnel IA	0.073	.02	✓	✓	—
	Perceived active control×tunnel IA	0.039	.09	✓	—	—
	Perceived personal relevance×tunnel IA	0.041	.01	✓	✓	✓
	Trust×tunnel IA	0.030	.11	—	—	—
	Perceived novelty×tunnel IA	0.005	.61	—	—	—
	User engagement×matrix IA	0.053	.09	✓	—	—
	Perceived active control×matrix IA	0.059	.02	✓	✓	✓
	Perceived personal relevance×matrix IA	0.008	.54	—	—	—
	Trust×matrix IA	0.025	.18	—	—	—
	Perceived novelty×matrix IA	0.006	.61	—	—	—

^a^IA: information architecture.

^b^Pathways indicated with a check mark were included in the model formulation.

^c^Pathways indicated with an em dash were excluded in the model formulation.

**Table 5:** Fit statistics of mediation models 2, 3, and 4.

Model	Chi-square (df)	*P* value	χ^2^ divided by df	CFI^a^	SRMR^b^	RMSEA^c^	95% CI
Model 2	4.7 (9)	.86	0.522	1	0.027	0.000	0.000-0.041
Model 3	10.8 (13)	.63	0.833	1	0.042	0.000	0.000-0.057
Model 4	14.7 (17)	.62	0.864	1	0.044	0.000	0.000-0.053

^a^CFI: comparative fit index.

^b^SRMR: standardized root mean square residual.

^c^RMSEA: root mean square error of approximation.

The model explains the effect of IA as follows: compared with hierarchical IAs, health information presented in a tunnel IA is perceived as more personally relevant (β=.18). This subsequently increases user satisfaction (β=.17). Matrix IAs, in comparison with hierarchical IAs, significantly increase the active control users perceive to have over the health information (β=.11), which also increases satisfaction (β=.27). Furthermore, the model shows that next to user perceptions of personal relevance and active control, user engagement and perceived trust in the health information affect users’ satisfaction with a patient education website. Although we hypothesized that perceived novelty would also be affected by IA and affect satisfaction and knowledge in turn, this was not the case. Finally, we already established that IA design did not directly affect knowledge acquisition. The model demonstrated that IA also did not indirectly influence knowledge, as none of the tested mediation pathways were significant. Knowledge acquisition was influenced by user engagement (β=.26), but user engagement itself was unaffected by IA.

### Serial Mediation by Perceived Control and User Engagement

**Figure 6:**
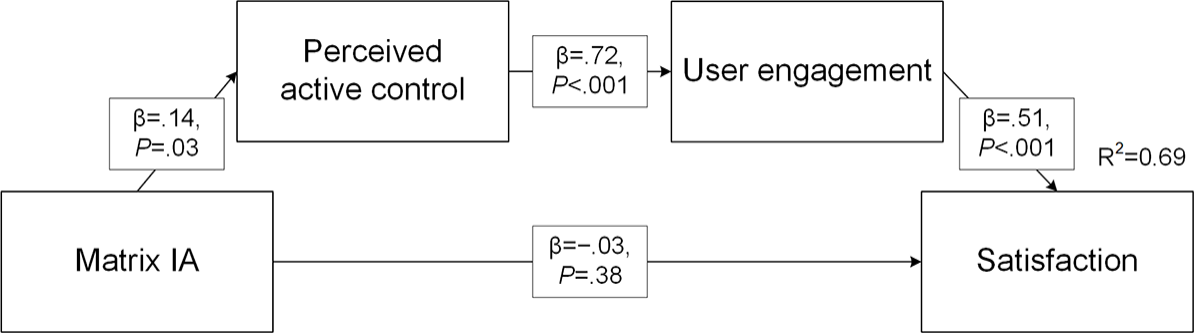
Serial mediation model of matrix information architecture effects on satisfaction via active control and engagement. IA: information architecture.

The serial mediation model, including perceived control and user engagement, confirmed that IA design did not significantly predict satisfaction (*P*=.07) or knowledge (*P*=.36). However, an indirect-only serial mediation by perceived control and user engagement on satisfaction emerged for matrix IA designs (β[indirect]=.052; *z*=2.053; *P*=.04) and hierarchical designs (β[indirect]=−.063; *z*=−2.545; *P*=.01), where matrix IA increased active control and subsequently user engagement and satisfaction, whereas hierarchical design decreased active control (Figure 6). Serial mediation was not present for tunnel IA (*P*=.65) or for knowledge (*P*_matrix_=.10, *P*_hierarchical_=.06, *P*_tunnel_=.65).

### Tailored IAs: Interactions With Patient Profile

**Figure 7:**
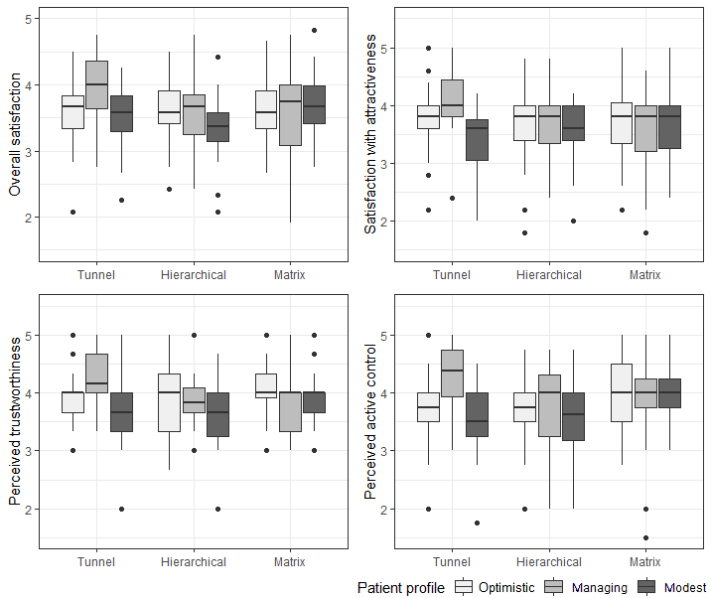
Interaction effects between information architecture and patient profile.

Interaction effects between IA and patient profile indicated that some IA designs were preferred more by users with specific profiles (*F*_4,206_=2.646; *P*=.04; η_p_^2^=0.049). In the post hoc analyses, a consistent difference was demonstrated between participants of the managing profile and modest profile using a tunnel IA design (Figure 7). Managing participants were significantly more satisfied with the tunnel design (mean difference to modest 0.489; *P*=.04), perceived it as more attractive (mean difference to modest 0.673; *P*=.01) and trustworthy (mean difference to modest 0.630; *P*=.009), and found it to provide more active control (mean difference to modest 0.764; *P*=.009).

## Discussion

### Principal Findings

The aim of this study is to investigate how the organization, display, and structural design of a website, that is, IA, influences patients’ experience with web-based patient education and the satisfaction and knowledge derived from the educational content. We wanted to understand whether user perceptions of engagement, control, personal relevance, trust, and novelty could explain how IA affects satisfaction and knowledge. Furthermore, we examined whether a user’s profile affected which IA design was most effective or enjoyable to explore the potential of tailored IA design. Research on IA in the context of web-based health education has been sparse [10], which has limited intervention designers’ ability to make informed design choices that enhance patients’ experiences with web-based education.

This study compared three IA designs: tunnel, hierarchical, and matrix design. We found that in comparison with hierarchical IAs, tunnel and matrix IAs slightly improve user satisfaction. This effect may be explained by increased user perceptions of personal relevance in the tunnel IA and increased perceptions of control in the matrix IA. Contrary to our hypotheses and earlier findings [11], no direct or indirect effects of IA on knowledge acquisition or website use were found. However, the findings did indicate that IA preferences differ between patients with different user profiles. Specifically, patients with a so-called *managing* profile, who prefer open communication and have high communicative capabilities, are more satisfied with health education that is presented in a tunnel IA.

Our finding that tunnel IA design specifically affects satisfaction with emotional support is consistent with research showing that tunneled education improved the emotional well-being of patients with type 2 diabetes and chronic low back pain [60]. However, we did not replicate previous findings indicating that tunneling increases the use of web-based health interventions [11,22]. We did perceive a trend in this direction: participants in the tunnel condition used the website longer on average. However, this difference was not statistically significant. IA design did not predict knowledge acquisition either, despite previous findings indicating that tunneling improves knowledge acquisition [11]. Instead, user engagement emerged as the only predictor of knowledge acquisition. Some research on patient education indicates that cognitive factors such as working memory and cognitive load may be better predictors of knowledge acquisition than the user experience variables included in this study [61]. As IA design may facilitate cognitive processes, for example, by presenting information in smaller chunks as done in the hierarchical and tunnel designs, exploring whether IA design influences cognitive factors may be a worthwhile avenue for future studies that could help explain a larger portion of the variance in knowledge acquisition. In general, knowledge acquisition scores were low (47%-52%), which is in contrast with earlier findings that show that web-based patient education is effective for orthopedic patients [62] even when websites are consulted just once [63]. However, we are unsure whether these findings are due to inadequate education offered or poor source material (which was not changed when converted from paper to website) or because we did not test knowledge before the experiment. Regarding the latter, if participants had very little knowledge of TJR to begin with, it may be that although attained knowledge levels were low, they still represented decent knowledge acquisition. The participants’ low self-reported knowledge of hip replacement supports this assumption: 81% of participants said that they had no or very limited prior knowledge. However, to fully answer this question, future research on IA design including pre-post measurements of health knowledge is needed.

The results of IA design on user engagement were mixed; the matrix IA achieved the highest subjective (ie, self-reported) engagement, but the tunnel IA was used the longest (albeit, not significantly longer). This reflects the dichotomous nature of engagement raised in the introduction, where engagement is thought to include both a subjective component of immersion and a behavioral component of use [15,64,65]. The findings indicate that IA design affects both but that matrix IA designs may be specifically suited for creating subjective experiences of engagement in patients. Furthermore, as only subjective self-engagement (and not duration of use) predicted actual knowledge scores, a very tentative conclusion may be that it might be more important to design engaging experiences rather than to design patient education materials that are used the longest. As most studies currently employ a *the more use, the better* perspective regarding engagement, use, and adherence to health interventions [66], this may require a different focus of researchers and designers alike.

Finally, this study focused on three simple IA designs for experimental clarity. Hybrid IAs that combine design elements from different IAs could mitigate the disadvantages associated with nonhybrid IAs. As users were most satisfied with matrix and tunnel IAs, hybrid matrix-tunnel designs should be explored further specifically. This study also identified that a large proportion of older adults with self-reported joint complaints use mobile phones (82%) and tablet devices (41%). As web-based IA designs cannot be ported to smartphones [13], IA designs suitable for health interventions distributed through mobile devices should be explored further. Finally, the field of IA has been affected considerably by the rise of recommender systems (RSs). These machine-based learning and information retrieval systems can predict and present relevant content, easing requirements for an adequate IA to help users locate content themselves. As this may diminish information overload [67], the potential benefits of combining RS techniques and IA in web-based health interventions warrant further research.

A secondary objective of this study is to explore the potential of tailored IAs. We found that participants with the highest information needs (so-called *managers*) preferred tunnel IAs. This finding supports the idea that patients’ web-based learning experiences may be improved when IA is tailored to relevant user characteristics. However, we did not envision beforehand that the tunnel IA would actually match the *managing* profile. Rather, we assumed that participants in this group would prefer a matrix IA, as their skills, high self-efficacy, and preferences for openness and participation are in line with the theoretical *ideal* user of matrix IA websites [12]. According to the qualitative feedback, one reason why they may have preferred the tunnel IA design instead is because it functioned as a checklist. Completeness or comprehensiveness is 1 of the 5 quality criteria for health information [68], and reassurance that all content had been covered may be particularly important for patients with high information needs. A tendency of patients with high information needs to actively seek out and ensure they have all available information (ie, a monitoring style of coping with threats) has been documented before in research with older patients with cancer [69]. Perceived comprehensiveness was not one of the mediators included in this study, but the question of whether some patients value it more than others, and which design elements may elicit comprehensiveness specifically, may be worthwhile avenues for future research. Finally, the patient profiles included in this study provided insight into orthopedic patients’ skills and preferences for general communication, not digital communication, specifically. Effective use of eHealth requires composite skills beyond basic literacy, such as being able to operate search functions and knowing what information is available on the web [70]. Therefore, it may be more accurate to tailor IA design to eHealth literacy levels instead of a general profile.

In any case, the incongruence between anticipated and actual match of patient profile and IA design indicates that translating stated preferences to a tailored design is complex. Although the knowledge base on what works for whom is growing slowly, it may be more beneficial in the meantime to offer users a choice of IAs rather than dictating one design. Studies that explored the benefit of tailoring the mode of health information (eg, text, illustrations, audio-visual material) have successfully used *user-initiated tailoring* when working with multiple interfaces [71,72]. User-initiated tailoring requests users to customize a website’s content and graphical user interface directly. Such customizations improve users’ satisfaction, users’ attention, and users’ ability to recall knowledge [71,72]. Possibly, user-initiated tailoring may also be applicable to tailored IA design if users are offered a choice of IA designs when they first visit the website. A second consideration is to design IAs that support many different styles of health information processing. The work by Pang et al [73] on a website that was purposely designed to support 4 (rather than 1) distinct health information-seeking behaviors showed that users were more engaged with these dynamic interfaces. The communality between these studies is that users were not restricted or coerced to use the website in a particular way but instead were able to customize the experience to their self-determined preferences and needs at the time of visiting. Although this design approach may improve the fit between user and design, it may also introduce new issues (such as motivating people to adjust interfaces) that warrant further research. Yet, as more intricate eHealth interventions are developed and examined, it should be taken into consideration that these findings show that none of the examined IA designs had serious negative effects on satisfaction and knowledge acquisition and that although advantages in terms of improved user experience were present, they were small. The added value of highly customizable interventions should, therefore, be examined in tandem with the additional costs associated with developing multiple interfaces.

### Strengths and Limitations

This study was conducted among adults who had self-reported joint complaints and may have viewed web-based education differently than patients scheduled for TJR surgery. However, previous studies have successfully tested health education websites in similar populations [11,71,72], and the high self-reported pain and lower health scores indicate that the study sample had considerable health concerns. As such, the sample can actually be considered a study strength as these individuals were likely motivated to learn about orthopedic health. At the same time, as the sample consisted solely of people with orthopedic health concerns, we know little of the generalizability of the findings of this study to other populations. Preferences for IA design may differ when using health education for purposes other than to prepare for TJR surgery (eg, to decide between alternative treatment options or to obtain support in managing a chronic illness), and additional research is needed to explore this.

Another limitation was self-selection, as participants were able to determine whether they wanted to join or leave the study. Between invitation for participation and inclusion in the study, 37% of participants were lost to follow-up. Of particular concern is that 6% of the sample dropped out after viewing the allocated website, as they might have done so based on their (negative) response to the website. This could make the study susceptible to type I errors [74,75]. This problem could not be remedied by intention-to-treat analysis due to the design of the experiment in which the participants that had dropped out generated no outcome data [75]. Therefore, we checked whether dropout was associated with allocation to a specific website, which was not the case. This made it unlikely that participants stopped because they were discontent with the allocated website. Another issue with self-selection was that participants could have been exceptionally interested in and already knowledgeable about TJR surgery. This would explain why we did not find any effects on knowledge. However, both self-reported prior knowledge of hip replacement and knowledge acquisition were generally low. A final limitation is that we determined satisfaction and knowledge gained from visiting the website once. As such, we cannot draw conclusions about experience with the website over time or knowledge retention after longer periods.

The strengths of this study include the experimental design. Although randomized experiments of website features known as *A/B tests* or web-based field experiments [76] are common in industry, the method is not often used in academic research on web-based health interventions. Various scholars have advocated moving beyond the *black box approach* which assesses only intervention efficacy. Testing specific features can help understand the mechanisms by which web-based interventions (do not) improve health outcomes [10,17,22]. By experimentally manipulating one feature and assessing both outcomes as well as mediating variables, this study takes a step in that direction. Second, the study took a human-centered and interdisciplinary approach to patient education design. The team included interaction designers, clinicians, and psychologists and followed an iterative design process that involved patients early via pilot studies to ensure the usability of all three variants of the website. We believe that this commitment to developing three distinct but comparable, usable, and enjoyable web-based experiences has made it more likely that the effects on satisfaction can be attributed to differences in IA alone.

### Conclusions and Recommendations for Intervention Design

Overall, our findings indicate that IA has small but notable effects on users’ experiences with web-based health education interventions, at least in the context of orthopedic patient education. Tunnel IA design, in which users are guided through sequentially ordered content, improves perceptions of personal relevance and, in turn, user satisfaction. This design may be specifically appropriate for patients with high information needs. In contrast, providing users with more control over the way they progress through a web-based health intervention via a matrix IA design has positive effects on user perceptions of active control, which also contributes to higher satisfaction. Although additional research on IA design in different target groups and interventions is needed, hierarchical IA designs are not recommended at the moment, as hierarchical content is perceived as less supportive, engaging, and relevant, which may diminish the use and, in turn, the effect of the educational intervention.

## Appendix

Multimedia Appendix 1 Dutch translation of the User Engagement Scale-Short Form: validity, questionnaire items, and instructions for scoring.

Multimedia Appendix 2 Perceived advantages and disadvantages of tunnel, hierarchical, and matrix information architecture designs (translated from Dutch).

Multimedia Appendix 3 CONSORT-eHEALTH checklist (V 1.6.1).

## Abbreviations

ANOVA: analyses of variance
CFI: comparative fit index
IA: information architecture
MC: multiple choice
RMSEA: root mean square error of approximation
RS: recommender system
SRMR: standardized root mean square residual
THP: total hip prosthesis
TJR: total joint replacement
UES-SF: User Engagement Scale-Short Form
