# Supplementary material for: Effects of Information Architecture on the Effectiveness and User Experience of Web-Based Patient Education in Middle-Aged and Older Adults: Online Randomized Experiment
Source: J Med Internet Res. 2021 Mar 3;23(3):e15846. doi: 10.2196/15846 (PMC7970227; doi:10.2196/15846)
Supplement: Multimedia Appendix 2 [file jmir_v23i3e15846_app2.docx]

**Multimedia Appendix B.** Perceived advantages and disadvantages of tunnel, hierarchical, and matrix information architecture (IA) designs (translated from Dutch).

| IA | Advantages | Disadvantages |
| --- | --- | --- |
| Tunnel | **Step-by-step approach is clear, legible, and provides a ‘checklist’**   - “I found it very clear, step-by-step in chronological order” - “the website was very nice and comprehensible and understandable for everyone. It is explained step by step what will happen, this is very clear!!” - “The step-by-step checking of how the process will go from start to finish when you have received a new hip” - “The information was clear and well-organized. No big pieces of text. The check marks on the left side of the page tell you which topics you can find information about.” | **Not being able to choose what you want to read**   - “I could not start with a specific part of information about the process myself.” - “I could not jump from one subject to the other; I did not try it extensively. But that is my impression now. You always had to click the arrows or next and could not click on a tab to read more.” |
|  | **Clear and simple navigation**   - “clear path, overview on the left” - “clear language and easy navigation” - “Easy to finish and very clear” | **High (initial) amount of information**   - “A lot of information” - “It seemed like a lot of information at the beginning; but later on it was not so bad.” |
|  | **Ability to revisit a previous topic**   - “Clear and structured, I can go back to each part if I want to” - “The most pleasant was the clear language and the ease with which you could return to the previous pages.” | **Repetitive**   - “I sometimes found the website somewhat monotonous” |
|  | **Transparency**   - “The clear explanation and the way it is kind of explained step by step, it is very transparent, from beginning to end.” |  |
| Hierarchical | **Concise, no unnecessary information**   - “Clear, short and concise” - “clear language, short and concise, well-organized” - “Very clear, large font for those who need it. Clear language. no unnecessary information” - “An understandable Dutch website. With no unnecessary information. Clear for when you are in such a situation.” | **Difficult to navigate to main menu when accessing deeply-nested information**   - “That "back to main menu" is below the text and therefore sometimes not visible. I prefer to have this [button] at the top.” - “The only thing that I found that, with the PC, that if you want to exit the menu, it was not clearly indicated to me.” |
|  | **Phases logically relate to the treatment itself**   - “Clearly arranged and divided into the different phases of treatment” - “I was able to view each phase in the treatment and healing process, grouped by subject at my own pace. There was a logical navigation and practical information was everywhere.” - “The choice menu, which shows stages that can be viewed, depending on the treatment, the complaints of the person afterwards. It answered all questions from shortly after and longer after the treatment. | **Fragmented information**   - “Some topics still contain relatively long text. I would find it much more pleasant if certain instructions are mentioned in a row, so that it is clearer sooner what you should do or should not do.” |
|  | **Knowing where you are and where you are going on the website**   - “The clarity of what you can read and the clarity that you know where you are on the website” - “where I could find everything” - “Clarity, you knew exactly where you were” | **Not knowing where you are going on the website**   - “that I did not always know where I was going.” |
| Matrix | **Clear, minimalistic overview**   - “Clear overview per theme” - “What you selected was very clear. You immediately got the relevant information.” - “I found that the boxes provided clarity and overview.” - “The clarity of the searchable information, on the first screen with all the topics together.” - “That you had clear buttons for all the different information. You just click the button and you get specific information. That better than an A4 with a lot of text in a row” - “Minimalistic, information is clearly clustered to boxes” | **Messy and chaotic**   - “It was messy; sometimes it was not clear where I ended und thus I was unnecessarily clicking back and forth” - “Although everything was discussed, the website also has something chaotic by ‘clicking’ the topics without any obligation. This would allow you to overlook something. I prefer to see a ’fixed’ order such as day of surgery, discharge, home, physiotherapy, checks, etc. and what you have to do or can expect during these occasions.” |
|  | **Rapid availability of specific information**   - “You can get the information quickly and easily just by pressing the buttons” - “The headings where clear so you know very quickly where to go if you want to know more about a specific topic.” | **Effortful and tiring**   - “I did not want to read all those headings…” - “I found the way to get information a bit cumbersome and messy with all those boxes. I think if you do not do a lot of computer work this will be tiring” |
|  | **Knowing where you are and where you are going on the website**   - “I knew exactly where I was and I could easily return if I wanted to read something again.” - “I always knew where I went” - “I liked the ability to click through, clear choice of topics so that you do not get lost when you want to read something again.” | **Boring**   - “The website looks a little boring. It is nice and minimalistic and clear, but rather boring.” |
|  | **Being able to choose what you want to read**   - “I liked that you could choose where and when you wanted to see information about something.” - “You can choose what you want to read and when. There is no determined order. It is clear.” |  |
